# Decreased IL-1 β Secretion as a Potential Predictor of Tuberculosis Recurrence in Individuals Diagnosed with HIV

**DOI:** 10.3390/biomedicines12050954

**Published:** 2024-04-25

**Authors:** Marina Nosik, Konstantin Ryzhov, Asya V. Kudryavtseva, Ulyana Kuimova, Alexey Kravtchenko, Alexandr Sobkin, Vitaly Zverev, Oxana Svitich

**Affiliations:** 1 I.I. Mechnikov Institute of Vaccines and Sera, 105064 Moscow, Russia; rkazaw@yahoo.com (K.R.); vitalyzverev@outlook.com (V.Z.); svitichoa@yandex.ru (O.S.); 2 Escuela de Medicina, Facultad de Ciencias Médicas, Universidad Bernardo O’Higgins, Santiago 8370993, Chile; kudassia@gmail.com; 3 Central Research Institute of Epidemiology, Rospotrebnadzor, 111123 Moscow, Russia; ulyanakuimova@gmail.com (U.K.); alexey-kravtchenko@yandex.ru (A.K.); 4 G.A. Zaharyan Moscow Tuberculosis Clinic, Department for Treatment of TB Patients with HIV, 125466 Moscow, Russia; alexandr@sobkin.net

**Keywords:** HIV infection, HIV/TB coinfection, tuberculosis recurrence, Il-1β, IL-10, INF-γ, TNF-α

## Abstract

**Background:** The mechanisms of the formation of immunological competence against tuberculosis (TB), and especially those associated with HIV co-infection, remain poorly understood. However, there is an urgent need for risk recurrence predictive biomarkers, as well as for predictors of successful treatment outcomes. The goal of the study was to identify possible immunological markers of TB recurrence in individuals with HIV/TB co-infection. **Methods:** The plasma levels of IFN-γ, TNF-α, IL-10, and IL-1β (cytokines which play important roles in the immune activation and protection against *Mycobacterium tuberculosis*) were measured using ELISA EIA-BEST kits. The cytokine concentrations were determined using a standard curve obtained with the standards provided by the manufacturer of each kit. **Results:** A total of 211 individuals were enrolled in the study as follows: 62 patients with HIV/TB co-infection, 52 with HIV monoinfection, 52 with TB monoinfection, and 45 healthy donors. Out of the 62 patients with HIV/TB, 75.8% (47) of patients were newly diagnosed with HIV and TB, and 24.2% (15) displayed recurrent TB and were newly diagnosed with HIV. Decreased levels of IFN-γ, TNF-α, and IL-10 were observed in patients with HIV/TB when compared with HIV and TB patients. However, there was no difference in IFN-γ, TNF-α, or IL-10 secretion between both HIV/TB groups. At the same time, an almost 4-fold decrease in Il-1β levels was detected in the HIV/TB group with TB recurrence when compared with the HIV/TB group (*p* = 0.0001); a 2.8-fold decrease when compared with HIV patients (*p* = 0.001); and a 2.2-fold decrease with newly diagnosed TB patients (*p* = 0.001). **Conclusions:** Significantly decreased Il-1β levels in HIV/TB patients’ cohort with secondary TB indicate that this cytokine can be a potential biomarker of TB recurrence.

## 1. Introduction

It is well known that tuberculosis (TB) is the leading cause of death among people living with HIV (PLHIV). According to the WHO, 703,000 people living with HIV contracted tuberculosis in 2021, of which about 187,000 died [1,2]. To date, a lot of clinical data have already been accumulated, indicating that *Mycobacterium tuberculosis* (*Mtb*) and HIV act synergistically, thus disrupting the body’s immune system, which leads to a subsequent increase in mortality in the absence of proper treatment [3,4,5]. Thus, people living with HIV are 16–18 times more likely to develop tuberculosis than people without HIV [1]. It is assumed that HIV infection causes a decrease in the number of macrophages capable of limiting the growth of *Mtb* [5]. Susceptibility to TB increases shortly after HIV infection, long before the number of CD4+ T cells decline to less than 500 cells/mL [5,6]. It is believed that, in people with latent TB, HIV infection is the most significant risk factor for tuberculosis, since HIV infection is highly likely to reactivate *Mtb* infection [7,8]. People living with HIV, regardless of the duration of ART, have a high risk of tuberculosis when compared to HIV-negative individuals [9]. In turn, TB also has a negative effect on the immune system when infected with HIV, accelerating the transition from HIV infection into AIDS [6,10]. It is suggested that since *Mtb* and HIV share anatomical reservoirs, such as the lungs, a favorable microenvironment is created in patients with TB, contributing to the development of HIV infection [6,11]. Constant immune activation in response to *Mtb* infections induces HIV replication in blood cells and at sites of *Mtb* infection in the lungs, as well as in activated cells of the pleural cavity [12,13,14]. This is due to the production of cytokines, which activate signaling transmission pathways in T cells and monocytic cells, which in turn leads to increased HIV transcription through the activation of transcription factors NF-kB (Nuclear factor-kB) and NFAT (Nuclear factor of activated T-cells) [5]. The differentiation of T cells in the foci of *Mtb* infections contributes to the fact that the expression of CCR5 and CXCR4 co-receptors increases on the surface of T cells, which makes these cells more susceptible to HIV infection since these co-receptors play a key role in the penetration of the virus into the cell [15,16,17,18,19,20]. According to WHO estimates, the mortality rate in HIV patients within 1 month after infection with *Mtb* in the absence of therapy is 90% [2]. People with HIV/TB co-infection have a high viral load even after recovery from tuberculosis when compared to people with HIV monoinfection [21,22]. At the same time, it should be kept in mind that the symptoms of tuberculosis associated with HIV infection are often non-specific [23]. Physicians note the difficulty of diagnosing TB in HIV-infected patients, especially at the stage of secondary diseases [23,24]. The atypical course of secondary tuberculosis in the late stages of HIV infection leads to an increase in the untimely diagnosis of TB. At the same time, the risk of TB recurrence must be taken into account. It is recognized that the key drivers of the high recurrence rate of TB include such factors as the fibrous-cavernous form of the disease, the destruction of lung tissue, positive sputum culture after 2 months of treatment [25,26], immunosuppressive conditions, and, in particular, HIV infection. Numerous clinical studies show that HIV infection is independently associated with a high risk of TB recurrence [27,28,29,30,31]. In regions with a high incidence of TB, the rate of TB recurrence among people living with HIV after standard treatments ranges from 14 to 37.7% [26,32,33]. Identifying individuals at high risk of TB recurrence prior to treatment could help in adjusting treatment regimens for such individuals, including the use of higher doses of drugs, additional drugs, or targeted therapies [34,35]. In this regard, the problem of detecting biomarkers that make it possible to identify among patients with HIV infection those at greatest risk of tuberculosis and a high risk of TB recurrence, even before the initial phase of therapy, is rather intense.

The immune response against pathogens such as HIV and *Mtb* is vigorous in acute infections, inducing the production of such pro-inflammatory cytokines as TNF-a, IFN-g, IL-1beta, and, later, after the enhanced secretion of those cytokines, anti-inflammatory cytokine IL-10 [36,37]. IFN-g controls a range of immunological and inflammatory responses in HIV infection, as well as in *Mtb* infection, and is deeply involved in the activation of monocytic cells [38,39,40]. In HIV infection, IFN-g plays a vital role in the reduction of HIV replication. It was shown that the progression of immunodeficiency was associated with diminished IFN-g production [40,41]. Also, IFN-g has a major role in protecting against TB [38,42,43]. The importance of IFN-g is confirmed by the fact that the incidence of TB in people with genetic abnormalities in the IFN-g expression increases dramatically [44]. TNF-α has a critical role in HIV infection by modifying HIV gene expression via the receptor-mediated activation of the HIV promoter region [45]. TNF-α upregulates HIV expression in T lymphocytes, macrophages, and monocytes by activating the NF-kB transcription factor [45,46,47]. It is suggested that TNF-α increases NF-κB’s binding to the HIV long terminal repeat, thus inducing viral replication. At the same time, TNF-α in HIV-1 infection mediates the death of HIV-infected cells due to apoptosis, and also inhibits HIV-1 replication in newly infected peripheral blood monocytes [46,48]. In *Mtb* infection, TNF-α plays a pivotal role in the granuloma formation, which is critical for controlling bacterial growth [8,49,50,51]. It is suggested that, in individuals with HIV/TB co-infection, *Mtb* accelerates HIV infection due to the specific formation of granulomas. It was shown that the granulomas formed in HIV-infected individuals were poorly formed and exhibited necrosis [45,52]. A marked decrease in TNF-α concentrations was associated with the formation of a defective granuloma [52]. IL-1beta is a key cytokine that induces host defense responses to infection by enhancing the antimicrobial properties of phagocytes and initiating Th1/Th17 adaptive immune responses [53,54]. It is shown that increased IL-1beta levels are associated with the progression of HIV infection, and, at the same time, the augmented secretion of this cytokine is vital for the control of *Mtb* infection [55,56]. Interleukin 10 (IL-10) is a key player in the establishment and persistence of viral infections. It is a multifunctional cytokine secreted by monocytes, macrophages, T cells, and dendritic cells, which can both induce or suppress the immune response [57,58]. This cytokine inhibits pro-inflammatory cytokine expression and antigen presentation, and blocks T-lymphocytes activation [58,59]. Its role in HIV infection is dual, as it can suppress HIV replication or promote virus production, depending on the IL-10 promoter polymorphisms and polymorphisms in chemokine receptors [60,61,62,63,64]. In *Mtb* infection, IL-10 is critical for downregulating the inflammation at the site of infection, reducing tissue damage via cell recruitment to the infected tissue [36].

Considering that both HIV and TB have a significant effect on the immune system, disrupting the normal balance of cytokines and the functioning of the cytokine network as a whole, a comparative study of the plasmatic level of cytokines (IFN-γ, TNF-α, IL-1 β, and IL-10) playing a crucial role in the regulation of protection against *Mtb* [8,45,49,55,65,66] was conducted in a group of patients with double HIV/TB infection (both newly diagnosed with TB and with recurrent TB), as well as in patients with HIV and TB monoinfections. The objective of this study was to identify potential markers of TB recurrence in people living with HIV.

## 2. Materials and Methods

### 2.1. Study Population

The subjects were recruited during 2019–2020 from different population pools at two large medical centers: the G.A. Zaharyan Tuberculosis Clinic and the Central Research Institute of Epidemiology. The subjects represented different population pools as follows: patients with dual HIV/TB infection (n = 62), patients with newly diagnosed TB infection (n = 52), and patients with newly diagnosed HIV (n = 52). Out of the 62 patients with HIV/TB, 47 (75.8%) patients were newly diagnosed with HIV and TB, and 15 (24.2%) patients displayed TB recurrence and were newly diagnosed with HIV. TB diagnoses were based on clinical symptoms, sputum microscopy, and radiological analyses. The patients were diagnosed as HIV-seropositive using ELISA (ARCHITECT HIV Ag/Ab Combo, Abbott Architect, Abbott GmbH, Wiesbaden, Germany; DS-IFA-AG/AT-SCREEN, NGO Diagnostic Systems, Nizhny Novgorod, RF; Invitrologic HIV - AG/AT- Ultra, Medical Biological Union LLC, Novosibirsk, RF; DS-IFA-AG- SCREEN, NGO Diagnostic Systems, Nizhny Novgorod, RF), and were then confirmed using Western blot (MilaBlot-HIV, NGO Diagnostic Systems, Nizhny Novgorod, RF). Healthy controls (n = 45) from the general population were recruited for the study at the blood transfusion center. Healthy controls (donors, HDs) were repeatedly tested negative for HIV-1 and had no history of TB or exposure to the disease within the past 6 months. At the time of enrollment, patients with HIV/TB were naive for ART and anti-tuberculosis therapy, HIV-positive patients were naïve for ART, and TB patients were naïve for antituberculosis therapy. In patients with TB recurrence, the second episode of TB occurred within 1.5–2.5 years following the initial successful TB treatment.

### 2.2. Ethical Statement

All individuals were over 18 years old and provided written informed consent for participation in the study. According to the General Data Protection Regulation (GDPR) requirements, all participants were deidentified and anonymized by assigning them unique codes, expressed as identifiers. All clinical samples, data, and study results were stored in an anonymized form. The study was conducted according to the guidelines of the Declaration of Helsinki and approved by the Biomedical Ethics Committee of the I.I. Mechnikov Institute of Vaccines and Sera (protocol #1/01/17/2018).

### 2.3. Sputum Microscopy and Culture

Sputum samples were stained for acid-fast bacilli, and were graded with light microscopy. The cultures were examined weekly for a maximum of eight weeks, or until positive for visible colonies.

### 2.4. CD4+ Cell Count

The CD4+ T-CELL count was carried out according to a standard procedure. The CD4+ T cell counting was performed with two-color flow cytometry using phycoerythrin-labeled anti-CD4 antibodies (FACSort, Becton Dickinson, Franklin Lakes, NJ, USA), according to the manufacturer’s instructions. The whole blood sample with anticoagulant was incubated with the fluorescent antibodies, and then the CD4+ cell number was determined with flow cytometry using Fluorescent Activated Cell Sorter BD FACSCount TM system (Becton Dickinson).

### 2.5. Cytokine Quantitation

The plasma levels of the cytokines IFN-γ, TNF-α, IL-10, and IL-1β were measured using the ELISA EIA-BEST Kit (Vector-Best, RF). Plasma was isolated according to the standard procedure. The whole blood was collected in a vacutainer with EDTA and centrifuged at 1000 rpm for 15–20 min with cooling. The plasma was collected, aliquoted, and stored at −80 °C until further analysis. The cytokine concentrations were determined using a standard curve obtained with the standards provided by the manufacturer with each kit (sensitivity 0–5 pg/mL), and the results were expressed as pg/mL.

### 2.6. Quantification of HIV-1 RNA

The quantification of HIV-1 RNA in blood plasma was performed via a real-time reverse transcription-polymerase chain reaction (RT-PCR) with the use of the “AmpliSens HIV Monitor-FRT” reagent kit (FSBI CRIE, Moscow, RF). The study was performed on the real-time PCR cycler Rotor-Gene Q (Qiagen, Hilden, Germany) using standardized technology with automated sample preparation. The analysis of the results was carried out using the software of the equipment.

### 2.7. Statistical Analysis

Comparisons of the variables between multiple groups were completed using Kruskal–Wallis tests. In instances where the Kruskal–Wallis *p* value was less than 0.05, indicating a difference between the groups, additional two-group comparisons were performed using Mann–Whitney U tests. The receiver operating characteristic (ROC) analysis was performed, and the area under the curve (AUC) was obtained for the potential marker by comparing the HIV/TB and HIV/TB recurrence groups. The data were analyzed using GraphPad Prism v9.5.0 (GraphPad Software, Boston, MA, USA) and STATISTICA 11.0 software (Tibco, Palo Alto, CA, USA). Multiple regression analysis was performed using the respective module of STATISTICA 11.0. Values of *p* < 0.05 were considered statistically significant.

## 3. Results

### 3.1. Study Population

A total of 211 individuals were enrolled in the study as follows: 47 patients with HIV/TB co-infection (HIV/TB); 15 displaying HIV/TB recurrence (HIV/TB-Rec); 52 patients with HIV monoinfection (HIV); 52 patients with TB monoinfection (TB); and 45 healthy donors (HD). The baseline characteristics of the study population are shown in Table 1. There were no significant differences in gender or age. There were no differences in cavities between the two groups of patients with HIV/TB and with TB alone. However, there were more patients with disseminated TB in the patients’ group with double infection, with 67–68% vs. 19.2% in the TB group, respectively. In both groups with HIV/TB co-infection prevailed patients with severe immunosuppression: 76.6% and 66.7% CD4+T cell count < 200 cells/mm^3^ vs. 30.8% in group with HIV alone (p = 0.0250). There was no difference in BMI (body mass index) or concomitant opportunistic infections between the two groups with double infection (Table 2).

There were no fatal outcomes during the studied period in any group.

### 3.2. Cytokines Distinguishing TB Recurrence in Individuals with HIV

**Figure 1:**
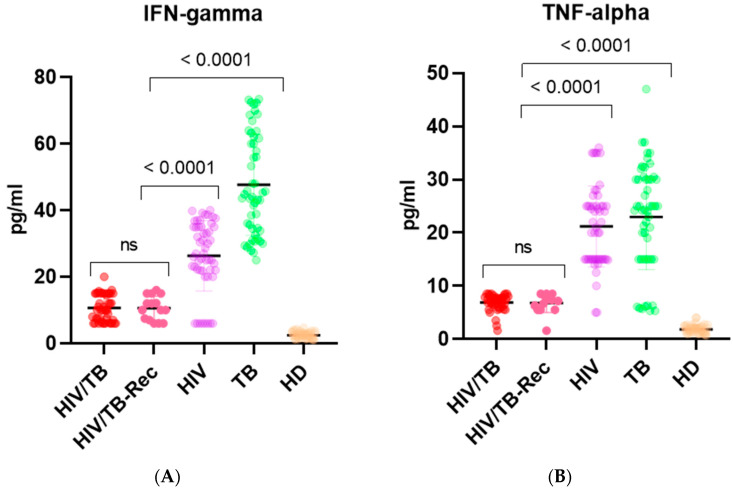

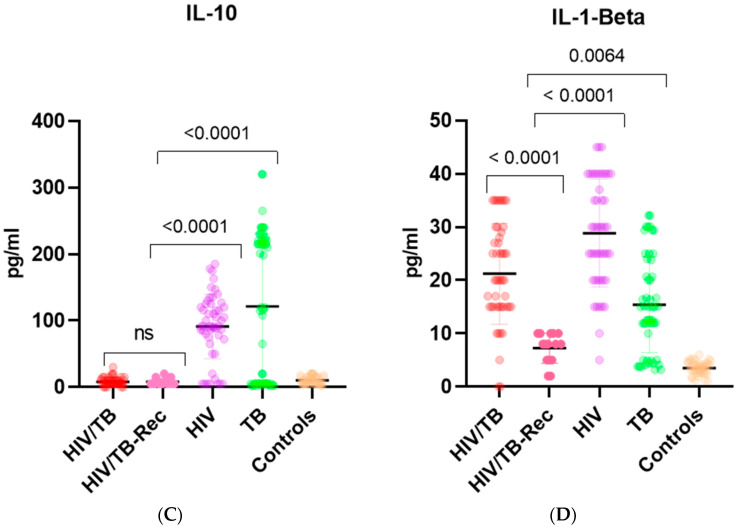
Plasma levels of cytokines in patients with HIV/TB co-infection, HIV/TB recurrence co-infection, HIV monoinfection, TB monoinfection, and controls. Interferon-γ (IFN-g) (**A**), tumor necrosis factor-alpha (TNF-alpha) (**B**), interleukin-10 (IL-10) (**C**), interleukin-1Beta (IL-1Beta) (**D**). Red circles designate the values of the group of HIV/TB co-infected; rose, HIV/TB patients with TB recurrence; lilac, HIV monoinfected; green, TB monoinfected patients; and beige, controls = HD. Statistical difference: Kruskal–Wallis test was used with multiple comparisons; *p*-value < 0.05 was considered significant.

The highest level of INF-γ production was observed in the group of patients with TB monoinfection, exceeding the levels observed in both patients with HIV monoinfection and with double HIV/TB infection (Figure 1A). In patients with HIV/TB, there was a 2.5-fold decrease in INF-γ production when compared to patients with HIV, and a 4.5-fold decrease when compared to patients with tuberculosis, respectively (*p* < 0.0001). The TNF-α levels in patients with HIV/TB co-infection were also significantly lower than in patients with HIV and TB monoinfection; cytokine production was reduced by 3.1 times and 3.4 times (*p* < 0.0001 and *p* < 0.0001, respectively; Figure 1B). At the same time, there was no statistically significant difference in the production of INF-γ and TNF-α in the groups of patients with HIV/TB and HIV/TB-Rec. A similar pattern was observed in the production of IL-10. The cytokine levels in patients with double infection were also reduced when compared to groups with HIV and TB alone; 11.1 times against the group with HIV monoinfection and 14.8 times against the group with TB monoinfection (*p* < 0.0001 and *p* < 0.0001, respectively; Figure 1C); however, between the two groups with HIV/TB, there was no difference in IL-10 production. However, in the group of patients with HIV/TB-Rec, there was a significant decrease in the production of IL-1β compared to the other three groups, especially with the HIV/TB group (Figure 1D). If, in patients with HIV/TB, IL-1β production was reduced 1.4 times (*p* < 0.0001) when compared with patients with HIV alone, then in patients with HIV/TB-Rec, cytokine production was reduced by 2.8 times (*p* < 0.0001) when compared with the group with HIV monoinfection and 3.9 times (*p* < 0.0001) when compared to the HIV/TB group. Multiple linear regression analysis showed that a statistically significant difference in cytokine production in the HIV/TB-Rec group was observed only for IL-1β (Table 3).

**Figure 2:**
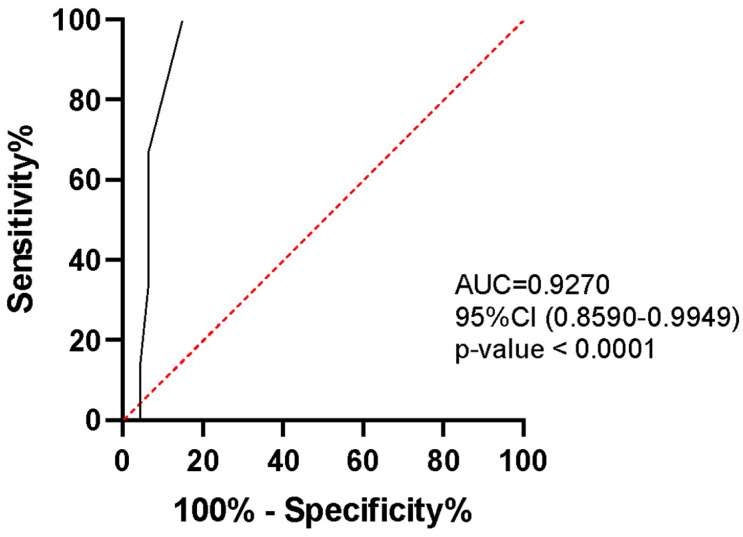
ROC analysis to estimate the IL-1β discriminatory significance of TB recurrence between patients with HIV/TB and HIV/TB recurrence.

In order to determine whether IL-1β can be considered a potential biomarker for predicting TB recurrence in HIV-infected individuals, a ROC analysis was performed. The ROC curve was obtained by comparing the HIV/TB group and the group with HIV/TB recurrence (Figure 2). Interleukin-1β showed significantly high AUC (0.9270, *p*-value < 0.0001), indicating that IL-1β could distinguish between the patients with HIV/TB and the patients with HIV/TB recurrence. 

A stringent analysis of the correlations of the cytokine levels with CD4+ cell counts did not reveal statistically significant differences between the two groups with HIV/TB co-infection (*p* values > 0.01).

## 4. Discussion

Cytokines play an important role in the immune response that determines the outcome of infections with intracellular pathogens. It is a well-known fact that both HIV and *Mtb* have a significant effect on the immune system, and both infections are characterized by a dysfunctional immune response [8,67,68,69,70,71,72]. HIV infection leads to a decrease in early immune control and a delay in the onset of adaptive immunity, which then results in a higher level of *Mtb* and, as a consequence, a greater bacterial load [73,74,75]. The important role of IFN-γ, TNF-alpha, IL-1β, and IL-10 as the cytokines essential for protection against *Mtb* is well established [8,45,49,55,59,65,75,76,77,78,79,80,81].

IFN-gamma plays a key role in the host defense against *M. tuberculosis* via activating the macrophages necessary for the production of reactive nitrogen species, in particular nitric oxide (NO), in order to restrict the growth of *Mtb* [59,76,82]. Furthermore, IFN-γ also plays a crucial role in several antibacterial processes, including granuloma formation and phagosome–lysosome fusion, which both cause the death of intracellular *Mtb* [49,76,82]. Many studies demonstrate that determining the level of IFN-γ can help in predicting active tuberculosis much earlier than the existing diagnostic algorithms. It has been shown that a high level of IFN-γ is a marker of the transition of TB from the latent stage to the active one, as well as the marker of early TB disease [77,83,84]. This is consistent with the data obtained in this study, during which a high level of IFN-γ production was detected in the group of patients with active TB, exceeding a similar indicator in the control group by 19.1 times, and 4.5 times in the groups with double infection (both *p* < 0.0001). We wanted to see if this indicator could be extrapolated in the case of TB recurrence in patients with HIV/TB co-infection. Here, in agreement with other studies, in patients with HIV/TB co-infection, we detected the decreased production of IFN-γ beyond the levels observed in both HIV and TB monoinfections (*p* < 0.0001), [85,86,87,88]. Previously, it was shown that low IFN-γ levels differentiated HIV/TB co-infection regarding the severity of clinical manifestations of TB [89]. This was confirmed by the results of this study, in which, in patients with double infection both with and without TB recurrence, a reduced level of IFN-γ has been correlated with a high percentage of severe forms of TB as follows: 48.4% of patients were diagnosed with disseminated TB, and 30.6% with infiltrative TB in the decay phase. However, no difference in IFN-γ production between the two HIV/TB groups was detected.

TNF-α, along with IFN-γ, play a significant role in the immunological and pathological reactions to tuberculosis (TB) via inhibiting the recurrence of TB and controlling the pathogenic response of the immune system and pulmonic expression of certain immunologic components [49,80,81]. TNF-α, interacting with other cytokines, and, in particular, with IFN-γ, induces a competent cell-mediated immune response to pathogens such as *M. tuberculosis* [90]. But, first of all, TNF-α has been identified as one of the key cytokines in controlling Mtb infection due to its role in the formation and maintenance of granulomas [51,80,91,92]. As the infection progresses, TNF-α coordinates the chemokine response within the lung and facilitates the development of the granuloma [49]. TNF-α has important pro-inflammatory functions, and low levels of this cytokine are associated with the progression of TB, right up to a fatal outcome. This is primarily a consequence of a decrease in the antimycobacterial reactions of macrophages simultaneously with a violation of the functionality of granulomas [93]. A number of studies have shown that TNF-α, in addition to IFN-γ, could serve as a diagnostic biomarker of TB infection since its production differed significantly between individuals infected with Mtb and those with no evidence of infection [77,94,95]. This was confirmed by the results of this study, where it was found that the level of TNF-α production in patients with TB monoinfection was 12.1 times higher (*p* < 0.0001) when compared with the control group. Nevertheless, we did not detect a statistical difference in TNF-α production between patients with HIV and TB monoinfections. The plasma levels of TNF-α were comparable in both groups with monoinfection, which is in line with other studies [51,85,86,87]. At the same time, in both patients’ groups with HIV/TB co-infection, the TNF-α levels were significantly decreased (on average by 3.3 times, *p* < 0.0001) when compared with groups with HIV and TB monoinfections. In one experimental model in vivo, it was shown that low TNF-α levels were associated with recurrent TB [96]. However, herein, as in the case of IFN-γ production, there was no statistical difference between the groups with TB recurrence and those without it.

There are data that the TNF-α/IL-10 and IFN-γ/Il-10 ratio may also serve as a reliable indicator of *Mtb* infection, as well as a marker that could distinguish between active TB and latent TB [95,97,98]. IL-10 is one of the regulatory cytokines that reduces inflammation and limits the activation of adaptive immune responses [99]. It is known that, at the initial stage of *Mtb* infection, when IL-10 levels are elevated, it acts as an inhibitor, downregulating the immune response and thereby limiting tissue injury [77,80,100,101,102,103]. However, the excessive production of this cytokine can lead to the failure of infection control and the reactivation of TB [80,101,102,104,105]. Herein, we, as well as other researchers, found an elevated expression of IL-10 in TB patients versus healthy subjects [103,106]. However, in both groups of patients with HIV/TB co-infection, that indicator was significantly decreased (on average by 14.8 times, *p* < 0.0001), and again there was no statistical difference in IL-10 production.

Thus, we observed no significant changes in IFN-γ, TNF-α, and IL-10 expression between the two groups with double infection. Interleukin-1β was the only cytokine that distinguished patients with HIV/TB and HIV/TB recurrence. Interleukin-1β is one of the powerful proinflammatory cytokines with pleiotropic activities [53,107]. It is known that IL-1β is necessary for the host’s control of mycobacterial infection [55,108]. Interleukin 1-β participates in the differentiation of naive T cells and affects the effector functions of various subpopulations of T and B lymphocytes. There are data that the IL-1β response is characterized by the inflammasome independence, which indicates that this cytokine may also be produced by an atypical cellular source [109]. It was shown that IL-1β is one of the cytokines critically required for host resistance, and that the absence of IL-1β could severely compromise the host response to *Mtb* [109,110,111]. Numerous data indicate that in the advanced stages of HIV monoinfection, as well as in *Mtb* monoinfection, there is an increase in the production of IL-1β [56,112,113]. Our findings are consistent with these reports. We detected a 4.5-fold increase in the IL-1β expression in the TB group and a 4.9-fold increase (both groups, *p* < 0.0001) in the HIV group when comparing with the reference values. On the contrary, the IL-1β production in both groups with HIV/TB co-infection was reduced, especially in the HIV/TB group with TB recurrence. The IL-1β levels in the HIV/TB-Recurrence group was decreased by 2.2 times, (*p* < 0.0064) compared with TB group; by 2.8 times, (*p* < 0.0001) vs. HIV group and by 3.9 times (*p* < 0.0001) vs. HIV/TB group. The strongest association of decreased IL-1β levels with TB recurrence was confirmed using multiple linear regression analysis, which makes it possible to consider this cytokine a potential marker. This is in concordance with another study, where it was showed ex vivo that lower IL-1β levels could serve as a predictor of TB recurrence [114].

The mechanism of IL-1β secretion is still poorly understood. However, the data accumulated suggest that several factors have a strong impact on its expression. IL-1β is produced as an inactive precursor of pro-IL-1β in response to the so-called pathogen-associated molecular patterns (PAMPs) [53,111,115]. The active form of IL-1β is formed as a result of the activation of inflammasomes, a multi-protein complex that stimulates the activation of caspase-1, thereby contributing to the production and secretion of proinflammatory cytokines [45,111,116]. After that, mature IL-1β is released into the external environment. The main producing cells of IL-1β are monocytes, macrophages, and dendritic cells [115]. However, for the transcription of IL-1β mRNA with the subsequent translation into pro-IL-1β protein, monocytic cells require the stimulation of TLR (Toll-like receptor) ligands, such as LPS (lipopolysaccharide) or NLR (Nod-like receptors) agonists [115,117]. Studies show that in the absence of an additional stimulus, cells release only a small amount of mature IL-1β into the extracellular environment [53,115]. Thus, the reduced expression of IL-1β may partly be explained by a lack of activating components. Also, another factor affecting the underexpression of IL-1β may be the depletion of the pool of cells secreting this cytokine. As a result of chronic immune activation caused by HIV and *Mtb*, cells function in an elevated mode, which then inevitably leads to their exhaustion [5,45]. In addition, CD4+ cell depletion may also be the result of pyroptosis. As mentioned earlier, the expression of IL-1β is triggered by two different triggers as follows: a signal that induces the expression of pro–IL-1β, and a signal that promotes the maturation of the IL-1β proteins via the activation of caspase-1 [109,118]. It has been shown that in HIV infection, caspase-1 triggers the pyroptosis of CD4+ cells [119,120,121]. Thus, if, in bacterial infections, pyroptosis contributes to the rapid elimination of pathogens via the elimination of intracellular replication reservoirs, then, in HIV infections, pyroptosis leads to an aggravation of the infectious process, thus contributing to the depletion of the CD4+ cell pool and chronic inflammation.

Another reason affecting the secretion of IL-1β may be the genetic diversity of *Mtb* strains. Numerous clinical data indicate that different strains of *M. tuberculosis* induce completely different immune responses [92]. In this work, we did not conduct a study concerning which *Mtb* genotypes the patients were infected with. But, for example, it has been shown that in the case of infection with *Mtb* strains belonging to the “not Beijing” genotypes, the inhibition of the basal proliferation of blood lymphocytes is observed, which inevitably leads to the reduced secretion of various cytokines [122]. And strains of *Mtb* Beijing are more virulent when compared to other *Mtb* genotypes, causing an unprotected immune response [92,123,124,125]. In vitro studies have shown that *Mtb* isolates associated with severe and moderate forms of TB induced a greater decrease in cytokine expression (including IL-1β) than in isolates obtained from patients with mild TB [126], which is evidence that the genetic diversity of *M. tuberculosis* affects aspects of host interaction with the pathogen, thus modulating immune responses.

Many studies have shown that a low CD4+ cell count might be one of the main predictors of TB recurrence in HIV-infected patients [26,30,33,127]. Nevertheless, in the present study, there was no statistical difference in the CD4+ cell number between the HIV/TB group and the HIV/TB group with TB recurrence. Both groups were distinguished by a low number of CD4+ cells as follows: 76.6% of patients in the HIV/TB group and 66.7% in the HIV/TB group with TB recurrence had a CD4+ cell count below 60 cells/mm^3^.

It is a well-established fact that smoking is one of the factors of TB recurrence, in addition to HIV infection [26,128,129,130,131,132]. However, this was not a decisive factor among the participants in this study, since the number of smokers among people with HIV and TB monoinfection was 92.3% and 82.7%, respectively, and, in both groups with HIV/TB co-infection, absolutely all (100%) participants smoked.

It is currently known that the incidence of TB recurrence can be mainly attributed to two mechanisms as follows: through a relapse of previous infections (endogenous reactivation), or through reinfection with a new isolate (exogenous reinfection) [6,133,134,135,136]. A relapse of the disease is defined as the second (or third) episode of active TB caused by the recurrence of the initial infection [135]. A mounting set of data show that, in HIV-negative individuals, most recurrences following successful TB treatment are due to endogenous reactivation [6,134,135,136,137]. Reinfection is a result of exogenous infection with a new *Mtb* isolate that differs from the isolate that caused the initial infection [135]. However, it should be noted that in high-TB endemic settings, in some cases, the individual could be exposed to reinfection with the same TB isolate that induced the primary infection [135,138]. Reinfection could happen in areas with high incidences of TB, as well as in settings with low to moderate incidences of disease [31,135,139,140]. These findings indicate that the initial *Mtb* infection does not protect against subsequent infection. Accumulated data show that relapses occur early following the end of TB treatment, usually within a year following therapy, while reinfection occurs after the first year following the treatment [134,135,141,142]. A growing body of evidence reveals that an increased incidence of TB recurrence in HIV-infected persons after the successful treatment of TB is due to exogenous reinfection, but not due to the relapse [6,134,136,140,142]. Moreover, it was demonstrated that 37.7% of patients with recurrent TB after being cured were HIV positive [32]. Lately, using molecular fingerprinting techniques, it is possible to differentiate between reactivation and reinfection [135,137,143], and whole genome sequencing (WGS) is the most preferred method due to its’ high discriminatory power [144,145]. However, this method is still quite expensive, limiting its use. We were unable to perform molecular genotyping in this work, but the data suggest that, among the HIV-infected participants of this study who had secondary TB, with a high degree of probability, we are referring to about exogenous reinfection. Indirectly, this is evidenced by the fact that, in all studies with recurrent TB participants, the time interval between the successful treatment of the first case of TB and a second episode of *Mtb* infection was 1.5–2.5 years.

## 5. Conclusions

In summary, we have shown that IL-1β was the only cytokine that distinguished the HIV/TB patients with TB recurrence from the HIV/TB patients. Neither IFN-γ, TNF-alpha, or IL-10 could differentiate between the two groups with double infection. We are aware of the limitations of this work associated with the small sample of patients (a limitation characteristic of several other studies in this field [114,146]), as well as the lack of opportunity to perform molecular genotyping. Future studies will be conducted for a wider group of patients and will include the fingerprinting of *Mtb* isolates. Nevertheless, the differences in the production of IL-1β between the two groups with HIV/TB co-infection with and without TB recurrence were large enough to identify statistical differences between those groups. Thus, the significantly decreased Il-1β levels detected in the cohort with HIV/TB patients than those with secondary TB indicate that this cytokine could be a potential biomarker of TB recurrence.

## Figures and Tables

**Table 1:** Baseline demographic and clinical characteristics of the study population.

Characteristics	HIV/TB (n = 47)	HIV/TB-Rec (n = 15)	HIV (n = 52)	TB (n = 52)	Healthy Donors (n = 45)
**Gender, (n/%):**					
**Male**	36 (76.6)	10 (66.7)	37 (71.2)	35 (67.3)	32 (71.1)
**Female**	11 (23.4)	5 (33.3)	15 (28.8)	17 (32.7)	12 (26.7)
**Age (years), IQR**					
**Male**	36.6 (25–55)	35.6 (30–42)	34.8 (26–54)	36.9 (28–59)	33.7 (21–45)
**Female**	35.8 (26–55)	33.3 (23–39)	34.5 (27–56)	33.5 (26–68)	33.4 (22–40)
**TB forms, (n/%):**					
**Disseminated**	32 (68.1)	10 (66.7)		10 (19.2)	
**Infiltrative**	15 (31.9)	5 (33.3)	_________	37 (71.2)	_________
**TB of intrathoracic lymph nodes**	_________	_________		5 (9.6)	
**CD4+ count (cells/mm^3^/%) IQR:**	202	208	279		
**<200**	59/76.6 (4–156)	54/66.7 (6–181)	56/30.8 (5–155)		
**<350**	226/23.4 (203–248)	222/33.3 (204–246)	259/50 (204–343)	545 (458–575)	895 (805–1450)
**>350**	_________	_________	395/19.2 (352–463)		
**Viral load (log_10_ copies/mL), IQR**	6.02 (4.41–7.0)	6.87 (4.59–7.0)	6.67 (3.9–7.6)	__________	_________
**Smoked (n/%):**					
**Yes**	47/100	15/100	48/92.3	43/82.7	n/a
**No**	_________	_________	4/7.7	9/17.3	
**Cavity (n/%)**	2/4.3	0	_________	3/5.8	_________
**Body mass index (BMI),**	20.8 (16.3–39.8)	20.0 (16–38.7)	n/a	n/a	n/a
**Time after 1st TB treated episode (years, n/%):**					
**1.5**	_________	2/13.3	_________	_________	_________
**2**		6/40			
**2.5**		7/46.7			

IQR—interquartile range; n/a—not available.

**Table 2:** Rate of opportunistic infections in individuals with HIV/TB co-infection.

Characteristics	HIV/TB (n = 47)	HIV/TB-Rec (n = 15)	*p*-Value
**Cytomegalovirus (CMV), n/%**	3/6.4	1/6.6	0.229
**Chronic hepatitis C/B virus (HCV/HBV)**	31/65.9	9/60.0	1.
**Herpes simplex virus (HSV)**	1/2.1	0	na
**Kaposis sarcoma**	0	0	na
**Candidiasis**	10/21.2	3/20.0	0.108
**Toxoplasmosis**	0	0	na

na—not applicable.

**Table 3:** Distinct profiles of cytokines production in patients with HIV/TB and HIV/TB-Recurrence through multiple linear regression analysis.

n = 62	b*	Standard Error of b*	b	Standard Error of b	T(99)	*p*-Value
**Intercept**			100.933	0.244074	497.822	
**IL-1β**	0.9366	0.088347	0.0394	0.006855	3.44891	0.039412
**IFN-γ**	0.77082	0.038262	0.0571	0.003417	2.0874	0.057138
**TNF-α**	0.002776	0.052191	0.0041	0.002889	1.4722	0.276703
**IL-10**	0.085931	0.04356	0.0038	0.001624	2.0760	0.378127

Regression Summary for Dependent Variable “TB Recurrence or not”: R = 0.87435818; R² = 0.76455317; Adjusted R^2^ = 0.74909907; F(5,58) = 33.348 *p* < 0.0000; Std. Error of estimate: 0.7834.

## Data Availability

Data available upon request.
